# Aldehydes as powerful initiators for photochemical transformations

**DOI:** 10.3762/bjoc.16.76

**Published:** 2020-04-23

**Authors:** Maria A Theodoropoulou, Nikolaos F Nikitas, Christoforos G Kokotos

**Affiliations:** 1 Laboratory of Organic Chemistry, Department of Chemistry, National and Kapodistrian University of Athens, Athens 15784, Greece

**Keywords:** aldehyde, green chemistry, photochemistry, photoinitiation, sustainable chemistry

## Abstract

Photochemistry, the use of light to promote organic transformations, has been known for more than a century but only recently has revolutionized the way modern chemists are thinking. Except from transition metal-based complexes, small organic molecules have been introduced as catalysts or initiators. In this review, we summarize the potential that (aromatic or aliphatic) aldehydes have as photoinitiators. The photophysical properties and photoreactivity of benzaldehyde are initially provided, followed by applications of aldehydes as initiators for polymerization reactions. Finally, the applications to date regarding aldehydes as photoinitiators in organic synthesis are presented.

## Introduction

Photochemistry, and especially photoredox catalysis have altered the way that modern researchers treat radical species [1–4]. In most cases, a metal-based photocatalyst is employed, having multiple advantages since ligand manipulation can lead to optimized photoredox properties. Unfortunately, the use of metals can pose some critical disadvantages in an organic process. Especially since the natural abundance of various noble metals that are used as photocatalysts is limited and since any pharmaceutically relevant compounds must not contain any metal contamination [5], the use of organic dyes is quite popular and can substitute the use of metal-based photocatalysts [6]. An alternative solution is the use of organic molecules as photocatalysts. There are already many reviews highlighting organocatalytically-mediated reactions [7–10] with potential in the field of photoorganocatalysis. The power of metal-based photocatalysts is indisputable and can be pinpointed through the ease that they can catalyze difficult photocatalytic reactions, such as the decarboxylation of aliphatic acids and the coupling of the residual chain with various electrophiles. Metal-based catalysts are common in reactions that require a high redox potential for a single electron transfer (SET) procedure to take place. On the other hand, even if organocatalysts have lower redox potentials, they are capable to induce other types of reactions, such as hydrogen atom abstraction (HAT) processes or triplet state energy transfer processes (EnT). Carbonyl compounds, especially diaryl ketones, have shown great potential as far as their catalytic scope is concerned. Benzophenone or acetophenone (**64**) and similar derivatives have already been used in a plethora of chemical transformations, and their photochemical properties have been studied extensively [11]. In an attempt to mimic the potential of ketones, various aldehydes have been employed as photocatalysts or photoinitiators. Even if aromatic ketones are useful photocatalysts, the corresponding aldehydes are not so widely utilized to promote chemical reactions. In this review, our goal was to summarize the photophysical properties of aromatic aldehydes, highlight the use of aldehydes as photoinitiators in polymerization reactions, and provide all examples where aldehydes were employed as photoinitiators in organic synthesis.

## Review

### Photophysical properties of carbonyl compounds

**Scheme 1:**
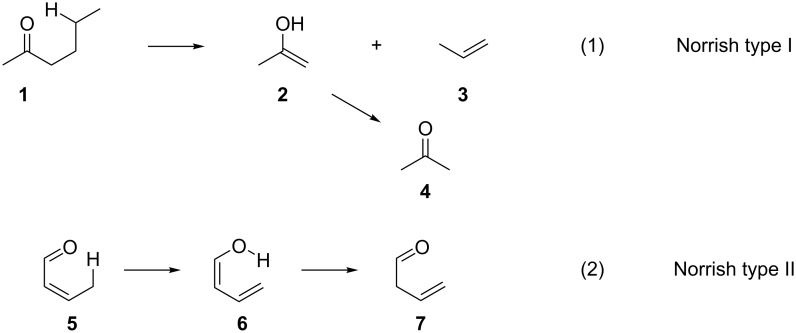
Norrish type I and II dissociations.

The interest in the interaction of aldehydes with light to promote reactions can be traced many years back. In 1966, Davies and co-worker studied the energy transfer from aliphatic ketones and aldehydes to olefins upon excitation of the carbonyl compound [12]. More specifically, aliphatic ketones and aldehydes can absorb irradiation in the long-wavelength region (240–340 nm), which is then transformed into electronic excitation through the S_1_ (n,π*) transition, excitation of a nonbonding electron on the oxygen atom to the first excited singlet state. This singlet state can lead to the corresponding triplet state T_1_ (n,π*) through intersystem crossing. This triplet state can be quenched through various chemical transformations, like Norrish type I and Norrish type II dissociations (Scheme 1). However, when a possible energy acceptor is present, the triplet state energy can be transferred from the carbonyl compound to the acceptor, returning the carbonyl compound to the ground electronic state. For example, in reaction (1) of Scheme 1, the β-bond dissociation of methyl *n*-butyl ketone (**1**) yields acetone (**4**) and propylene (**3**). Reaction (2) of Scheme 1 refers to a type II intramolecular rearrangement of crotonaldehyde (**5**) to 3-butenal (**7**). The triplet state energy donors were quenched by diacetyl and *cis*-2-butene.

**Scheme 2:**
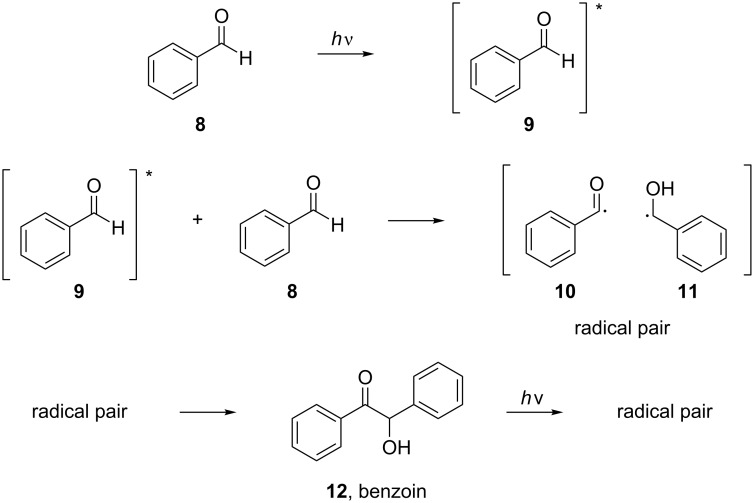
Proposed radical pair formation after the photolysis of benzaldehyde (**8**).

In 1970, Cocivera and Trozzollo studied the photolysis of benzaldehyde (**8**) in solution by NMR [13]. After recording the NMR spectra of ground-state benzaldehyde (**8**) and the derived products after irradiation, they were able to detect short-living paramagnetic intermediates, species with unpaired electrons, which, although existing in low concentration, could be detected because the unpaired electrons strongly polarized the nuclear spins. This type of polarization is retained when the intermediates return to their ground state. In order to account for the recorded spectrum, a radical pair formation between an excited molecule of benzaldehyde (**9**) and a ground-state molecule of benzaldehyde (**8**) was proposed. The excited molecule of benzaldehyde (**9**) could dissociate to a benzoyl radical (**10**) and a hydrogen atom or to a radical pair of a benzoyl radical (**10**) and an α-hydroxybenzyl radical (**11**), yielding a molecule of benzoin (**12**), which could be cleaved again upon irradiation, reforming the radical pair (Scheme 2).

**Scheme 3:**
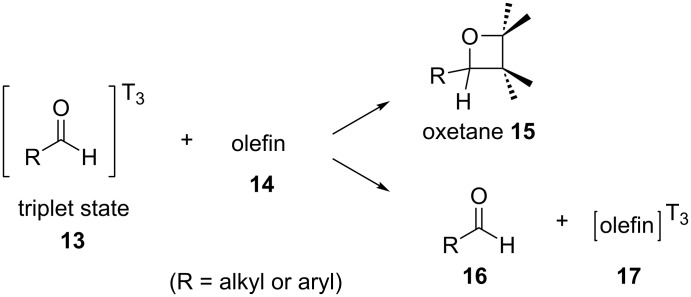
Aldehydes in the Paterno–Büchi reaction.

At the same time, Closs and Paulson came to the same conclusions via chemically induced dynamic nuclear spin polarization (CIDNP) NMR studies of an irradiated solution of benzaldehyde (**8**) [14]. Interestingly, around the same time, Yang and co-workers showed that aldehydes can transfer their triplet state energy to an acceptor, inducing changes in the reactivity of the acceptor. Accordingly, the aldehyde can react with the higher-energy-state olefin via a Paterno–Büchi reaction, indicating that benzaldehyde (**8**) can have an EnT ability comparable to that of known photocatalysts, such as aryl ketones (Scheme 3) [15–17].

**Scheme 4:**
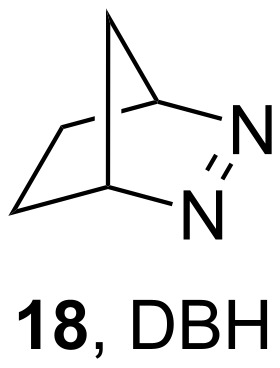
2,3-Diazabicyclo[2.2.1]hept-2-ene (DBH).

Later, Steel and co-workers examined the photochemical properties of benzaldehyde (**8**), studying the EnT from **8** to azo compounds [18]. Upon the irradiation and excitation of benzaldehyde (**8**), the phosphorescence was quenched either by the formation of the radical pair mentioned in Scheme 2 or by a triplet state-energy acceptor, such as 2,3-diazabicyclo[2.2.1]hept-2-ene (DBH, **18**, Scheme 4).

Trying to shed more light on the photolysis of benzaldehyde, Atkins and co-workers studied the photolysis of benzaldehyde (**8**) with electron spin resonance flash photolysis (ESRFP) and CIDNP in various solvents, depending on the efficiency of the solvents as hydrogen donors [19]. More specifically, triplet state benzaldehyde (**9**) dissolved in an efficient hydrogen donor solvent can lead to an α-hydroxybenzyl radical (**11**) as the only detectable species, which will rapidly react with the solvent. In nondonating solvents, such as hexane, triplet state benzaldehyde (**9**) reacts with ground state benzaldehyde (**8**) to form a radical pair of a benzoyl radical (**10**) and an α-hydroxybenzyl radical (**11**). The formation of benzoin (**12**) was also detected in solvents that did not react with the excited benzaldehyde (**9**, Scheme 2).

**Scheme 5:**
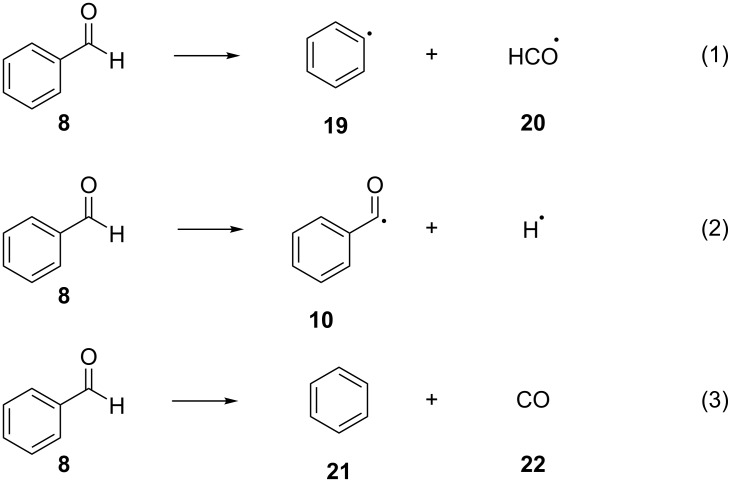
Dissociation pathways of benzaldehyde.

Photoorganocatalysts are known for their ability to abstract hydrogen atoms from various substrates, furnishing radical species. Benzophenone is a very well-studied example of this category. Alongside with benzophenone and acetophenone, benzaldehyde (**8**) has also been studied for the ability to carry out HAT processes. In 1975, Obi and co-worker studied the photochemistry of excited benzaldehyde (**9**) with the use of electron paramagnetic resonance (EPR) [20]. They detected that the generated radicals from this process were the α-hydroxybenzyl (**11**) and the benzoyl radical (**10**). Also, their relative abundance showed that the α-hydroxybenzyl radical (**11**) was formed faster than the benzoyl radical (**10**), with the latter being probably a product of the direct dissociation of **9**. They suggested that benzaldehyde (**8**) and related compounds, such as aryl ketones, could participate in HAT processes as hydrogen atom abstractors [20]. More specifically, aldehydes can absorb irradiation at 300 nm and be excited from their ground state S_0_ to their singlet state S_1_ (n,π^*^), Then, through intersystem crossing (ISC), they can transition to a lower energy state Τ_2_ (π,π^*^), which will subsequently drive them to a lower triplet state T_1_ (n,π*), with the latter one being responsible for the specific reactivity of the carbonyl compounds [21–22]. During the photoexcitation of a carbonyl compound, and after they reach the T_2_ excited triplet state, there are three possible dissociation pathways that can be followed (Scheme 5). Moreover, Reilly and co-workers observed the decomposition of benzaldehyde (**8**) into benzene (**21**) and carbon monoxide (**22**) (reaction (3) in Scheme 5) after the irradiation at 258.9 nm via laser ionization mass spectrometry and photoelectron spectroscopy [23].

**Scheme 6:**
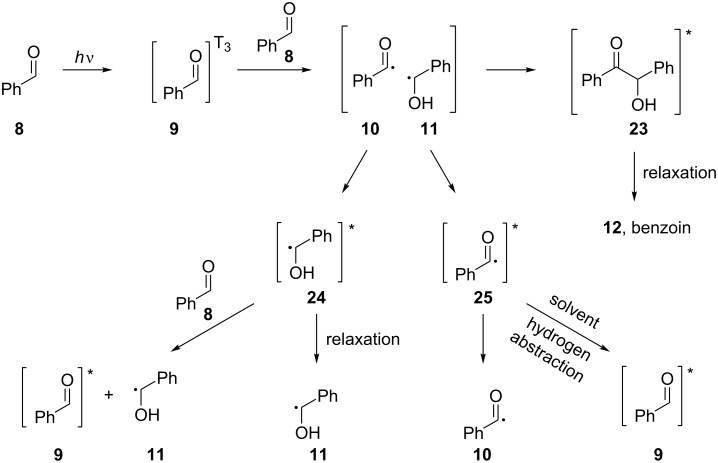
Reactions that lead to polarized products detectable by CIDNP.

In 1975, MacLauchlan and co-worker carried out a quantitative CIDNP study on the photolysis of benzaldehyde (**8**) in solution. They presented a possible pathway for the formation of the various radical species (Scheme 6) [24].

It was suggested that the triplet state of benzaldehyde (**9**) can abstract a hydrogen atom from a molecule of the solvent, furnishing a solvent molecule radical, which can propagate the radical chain route.

The photophysical properties of benzaldehyde (**8**) were thoroughly examined, allowing the researchers to better understand the source of this specific reactivity [25]. The triplet state energy of carbonyl compounds can also be transferred to halogen-containing compounds, leading to the homolytic dissociation of the carbon–halogen bond. This interaction has been studied for *tert*-butyl chloride, where the descendant radical is quite stabilized [26]. The electronic distribution and the spectroscopy of the excited states of benzaldehyde (**8**) have been examined. These studies illuminate the ways that the various excited states of benzaldehyde (**8**) decay, either through phosphorescence or through the dissociation to benzene (**21**) and carbon monoxide (**22**) or to a benzoyl radical (**10**) and a hydrogen atom (reactions (2) and (3) in Scheme 5). A practical application of these studies can be seen in the work of Kuhn et al., who demonstrated the use of the dissociation properties of benzaldehyde (**8**) and phenylglyoxylic acid in order to achieve highly deuterated products, which were more stable than the undeuterated ones upon irradiation and subsequent excitation [27–28]. The excited triplet state T_1_ (n,π^*^) of carbonyl compounds tends to be the most reactive state (compared to the singlet state). Furthermore, this excited state is easily accessible, as the quantum yield for the formation of the triplet state is close to unity, as measured by Itoh [29].

The dissociation route depends on the reaction conditions. Recently, Zhu and Cronin studied the dissociation of benzaldehyde after photoexcitation at 280–320 nm. The most efficient wavelength was found to be 285 nm, and the followed pathway was the pathway described in reaction (1) of Scheme 5 [30]. In another study on the photochemical dissociation of benzaldehyde (**8**), Bagchi et al. detected two major routes, furnishing either a formyl radical (**20**, reaction (1) of Scheme 5) or carbon monoxide (**22**, reaction (3) of Scheme 5) upon photolysis at 248 nm and 265 nm, respectively, while upon irradiation at 193 nm, reaction (1) from Scheme 5 was the main pathway [31]. The main reason for not observing the reaction (2) from Scheme 5 was that the product, the benzoyl radical (**10**), could decompose rapidly upon irradiation, supporting the other two pathways. Also, mechanistic studies by Cui et al. showed that the energy levels of the reactions (1) and (2) in Scheme 5 were closely tied, so distinguishing them is not always trivial [32].

The triplet state quenching of **8** and related compounds, such as aryl ketones, has been studied and mentioned numerous times. More specifically, Turro and co-workers studied them with time-resolved electron spin resonance and laser flash spectroscopy. The photolysis of **8** showed that the fate of the triplet state of **8** was also dependent on the concentration of **8** and on the type of the solvent [33]. Where the concentration of the aldehyde **8** is relatively high and the solvent is a poor hydrogen donor, such as acetonitrile, the HAT process from the solvent will not occur. In this case, a self-reduction of the excited benzaldehyde (**9**) will occur, leading to the formation of a radical pair of an α-hydroxybenzyl radical (**11**) and a benzoyl radical (**10**). This upper statement shows the potential of benzaldehyde (**8**) compared to the related aryl ketones, which are known to be capable of HAT.

Until recently, the quenching of the fluorescence of excited benzaldehyde (**9**) was unreasonably thought to originate from the basicity of the aldehyde. A recent publication though came to empower the statement that the HAT properties of benzaldehyde (**8**) are responsible for the observed quenching of the fluorescence [34].

### Aldehydes as photoinitiators for photochemical polymerizations

**Scheme 7:**
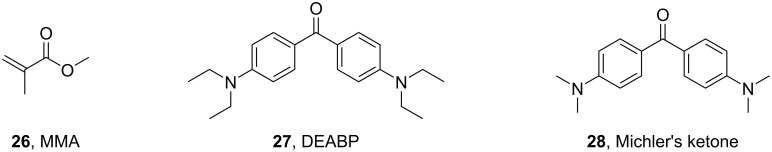
MMA (**26**), DEABP (**27**), and Michler’s ketone (**28**).

A number of carbonyl compounds, including aliphatic or aromatic ketones and aldehydes, have been employed as photoinitiators in polymerization reactions. The first studies reported in the literature where carbonyl compounds were used as initiators inspired researchers to study the use of aldehydes as well. In 1978, McGinniss and co-workers reported the polymerization of methyl methacrylate (**26**, MMA) by employing 4,4’-bis(*N*,*N*-diethylamino)benzophenone (**27**, DEABP) as the photoinitiator (Scheme 7) [35]. It was already well-accepted that aminoaromatic compounds, such as Michler’s ketone (**28**) and DEABP (**27**), present large extinction coefficients and charge transfer states as well as relatively long-lived triplet states in solution, making them suitable as photoinitiators [36–37].

**Scheme 8:**
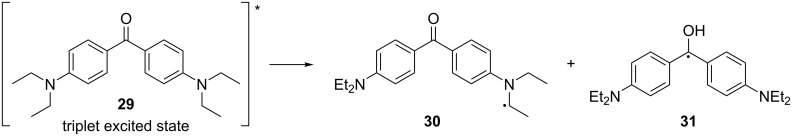
Radical intermediates of DEABP.

The irradiation and excitation of DEABP (**27**) to the singlet state is followed by intersystem crossing to the triplet state **29**, which then leads to two different types of free radical intermediates, **30** and **31** (Scheme 8). The species **31** was shown to be the primary initiating radical compound.

**Scheme 9:**
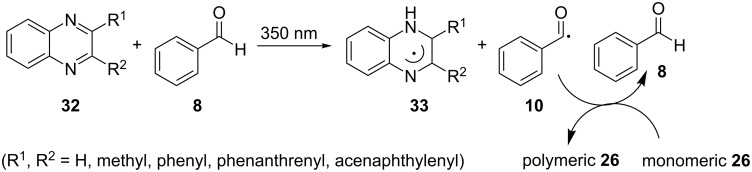
Photoinitiated polymerization of monomeric MMA (**26**) using the quinoxalines **32** and benzaldehyde (**8**).

In 2006, Aydin and Arsu employed benzaldehyde (**8**) as the coinitiator for the photoinitiated free radical polymerization of multifunctional MMA (**26**) [38]. Benzaldehyde (**8**) was tested as an alternative to tertiary amines, which are commonly employed in radical-mediated reactions due to their ability to bind to atmospheric oxygen, which inhibits such reactions. The quinoxalines **32** were used as the prime photoinitiators, and the reaction was placed in a photoreactor equipped with a cooling fan and lamps emitting at 350 nm at room temperature. The polymerization reaction led to good results. As depicted in Scheme 9, the postulated reason for the successful polymerization was that after the irradiation of benzaldehyde (**8**) with the quinoxalines **32**, the benzoyl radical (**10**) was formed from **8**. This was thought to be the initiating radical for the photoinitiated polymerization of monomeric MMA (**26**).

**Scheme 10:**
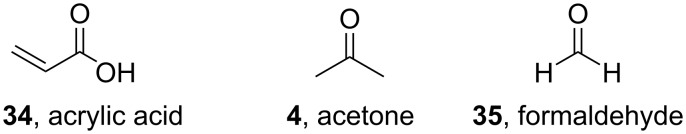
Acetone (**4**) and formaldehyde (**35**) as photografting initiators.

The photochemical properties of aliphatic aldehydes have also been employed in photografting. Even if they are not the most efficient photoinitiators, aliphatic aldehydes and ketones were studied in the photografting of acrylic acids. In 1988, Allméar and co-workers, while studying the grafting of acrylic acid (**34**) onto high- or low-density polyethylene and polystyrene using benzophenone as the photoinitiator and acetone (**4**) as the solvent, observed that the grafting was possible even in the absence of benzophenone due to acetone (**4**), but at a slower rate [39]. Later, Kubota and co-workers found that acetone (**4**) could act as an efficient photoinitiator for photografting, in contrast to other aliphatic ketones [40]. In 2004, Wang and co-workers also observed that acetone (**4**) could efficiently initiate photografting under UV irradiation when mixed with water [41] and performed a theoretical study on the matter a few years later [42]. The same research group later studied other aliphatic ketones for their efficiency in initiating photografting [43–44]. Recently, they reported that formaldehyde (**35**) could also act as a photoinitiator when employed for photografting even though it was not as efficient as acetone (**4**) (Scheme 10) [45].

**Scheme 11:**
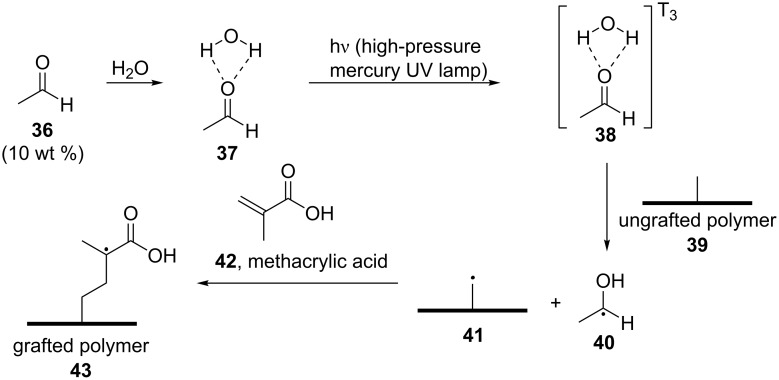
Photografting by employing acetaldehyde (**36**) as the photoinitiator.

The proposed mechanism of action is depicted in Scheme 11. The same research group extended their study and found that acetaldehyde (**36**) was also capable of initiating the photografting of methacrylic acid (MAA, **42**) onto HDPE, highlighting that acetaldehyde (**36**) is a better initiator that outperformed formaldehyde (**35**) and acetone (**4**) [46].

In more detail, in 2011, Wang and co-workers studied the efficiency of acetaldehyde (**36**) as the photoinitiator for the photografting of MAA (**42**) onto high-density polyethylene (**39**) in an aqueous solution [46]. The light source was a high-pressure mercury UV lamp (2 kW), and a cooling fan was used in order to maintain ambient temperature. Acetaldehyde (**36**) proved to be more efficient than acetone (**4**) and formaldehyde (**35**) in initiating the photografting in an aqueous solution. The maximum extent of grafting was observed for 10 wt % of **36**. The concentration of **42** also affected the extent of photografting. The extent of grafting increased with the increase of the monomer up to 2 mol/L and then kept constant or slightly decreased. The possible initiation of the polymerization is presented in Scheme 11, where hydrogen bonding of **36** with water is crucial.

**Scheme 12:**
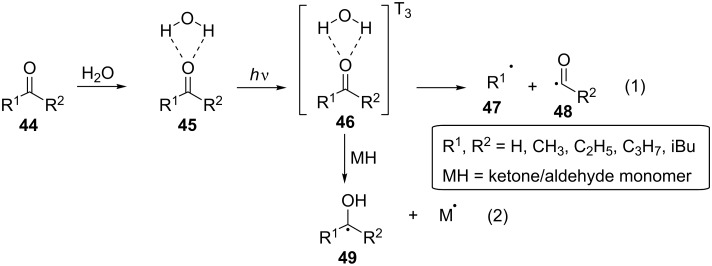
Proposed photolysis mechanism for aliphatic ketones **44** and formaldehyde (**35**).

In 2013, Wang and co-workers also reported the use of aliphatic ketones and aldehydes as photoinitiators for the photopolymerization of **42** in aqueous solutions [47]. For the cases where the ketones or the aldehydes were not soluble in, or miscible with water, an amount of ethanol was added. The light source employed was a UV high-pressure mercury lamp (2 kW). All aliphatic ketones tested, including acetone (**4**), butanone, and pentan-2-one, as well as formaldehyde (**35**), efficiently initiated the polymerization of MAA (**42**), and butanone was the most efficient compound. Since acrylates and methacrylates can be photopolymerized by self-initiation, a blank experiment without the use of any aliphatic ketone **44** or formaldehyde (**35**) was carried out, indicating that the polymerization of MAA (**42**) was mainly induced by photoinitiation by the aliphatic ketones **44** or formaldehyde (**35**), rather than by self-initiation of the monomer. Furthermore, oxygen was shown to strongly inhibit the photopolymerization, and an increase in the UV intensity increased the percentage of conversion and the polymerization rate. Finally, the percentage of conversion decreased, whereas the polymerization rate increased with an increase of the initial monomer concentration since an increase in the concentration of the monomer led to MAA (**42**) absorbing more energy than acetone (**4**). The authors proposed that the photoinitiation mechanism for the photopolymerization of aliphatic ketones or aldehydes was similar to the one concerning photografting, and the mechanism is presented in Scheme 12 [42,44]. While the aliphatic ketones **44** did not exhibit photoinitiation when they were alone present in an aqueous solution, they tended to form hydrates **45** with water via hydrogen bonding. The hydrogen bonding increased the energy levels of the excited states of **45**, permitting the formation of radicals after UV light absorption [48]. These radicals either dissociated via Norrish type I reactions, forming two radicals, **47** and **48** (reaction (1) in Scheme 12), or abstracted a hydrogen atom from a ketone/aldehyde monomer, forming a monomeric radical and an alcohol radical **49** (reaction (2) in Scheme 12). The excited state of the hydrates **45** and **46**, in an aqueous solution may prefer to abstract a hydrogen atom from a ketone/aldehyde monomer molecule rather than to decompose, forming a monomeric radical, which can initiate the polymerization.

**Scheme 13:**
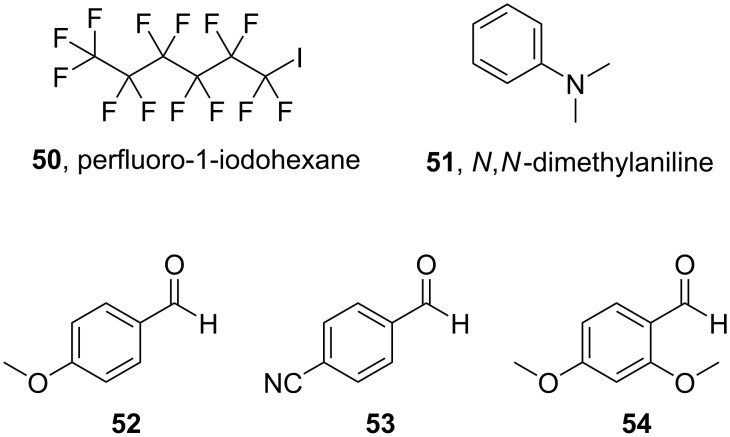
Initiator **50**, reductant **51**, and benzaldehyde derivatives **52**–**54** for the polymerization of the methacrylates **59**.

In 2017, Ma and co-workers performed a photoinduced controlled radical polymerization of methacrylates using perfluoro-1-iodohexane (**50**) as the polymerization initiator and benzaldehyde derivatives as organic photocatalysts [49]. 23 W compact fluorescent lamps were employed as the light source and *N,N*-dimethylaniline (**51**) was used as a potential reductant. The benzaldehyde derivatives tested are presented in Scheme 13 and included 4-anisaldehyde (**52**), 4-cyanobenzaldehyde (**53**), and 2,4-dimethoxybenzaldehyde (**54**), all three of which showed strong absorption bands below 340 nm.

**Scheme 14:**
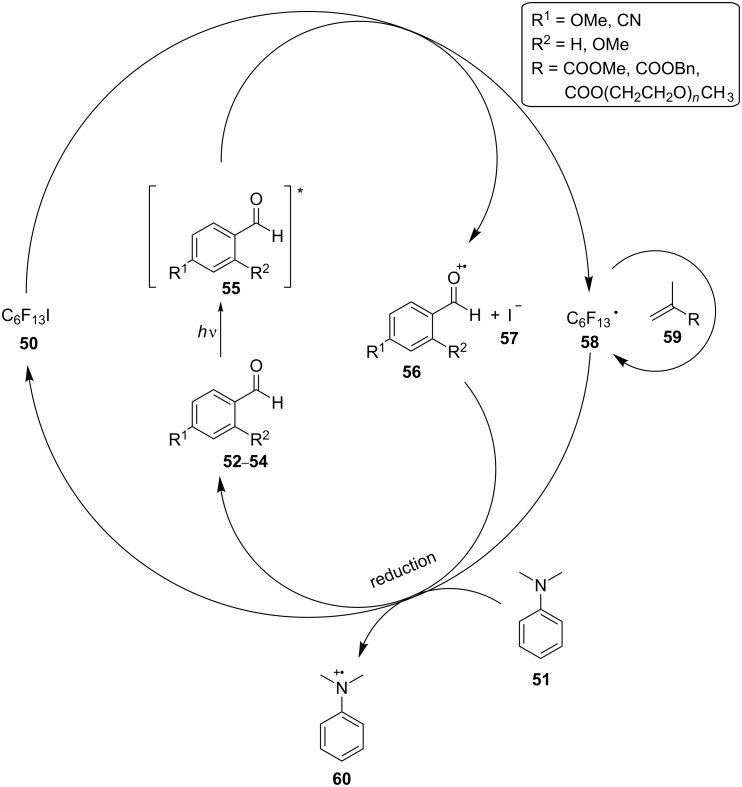
Proposed mechanism of the photomediated atom transfer radical polymerization employing the benzaldehyde derivatives **52**–**54**, respectively, as the photocatalysts.

As shown in Scheme 14, the excited state **55** of the benzaldehyde derivatives **52**–**54**, respectively, that occurred after irradiation could reduce perfluoro-1-iodohexane (**50**), generating a benzaldehyde radical cation (**56**) and the radical of the alkyl group C_6_F_13_, **58**, which was proposed to initiate the radical polymerization of a methacrylate monomer **59**. *N*,*N*-Dimethylaniline (**51**) acted as the reducing agent, regenerating the ground state of the benzaldehyde catalysts **52**–**54**, respectively, establishing an equilibrium between the dormant species and the active radicals, which was required for a controlled radical polymerization.

No polymerization product was detected in the absence of the benzaldehyde derivatives **52**–**54**, respectively, as well as when the polymerization was performed in darkness. Furthermore, the authors studied the effect of the concentration of the benzaldehyde derivative, the initiator, and *N*,*N*-dimethylaniline (**51**) and the molar ratio of the compounds.

### Aldehydes as photoinitiators in organic synthesis

The knowledge of the photophysical properties of aldehydes and the ability of aldehydes to initiate chemical transformations after irradiation was the first milestone in using them as initiators in organic synthesis. In the meantime, the use of aldehydes as reagents in some chemical transformations that employ a light source for photoexcitation shed more light on some mechanistic pathways and set the key foundation for the further use as photoinitiators or photocatalysts.

**Scheme 15:**
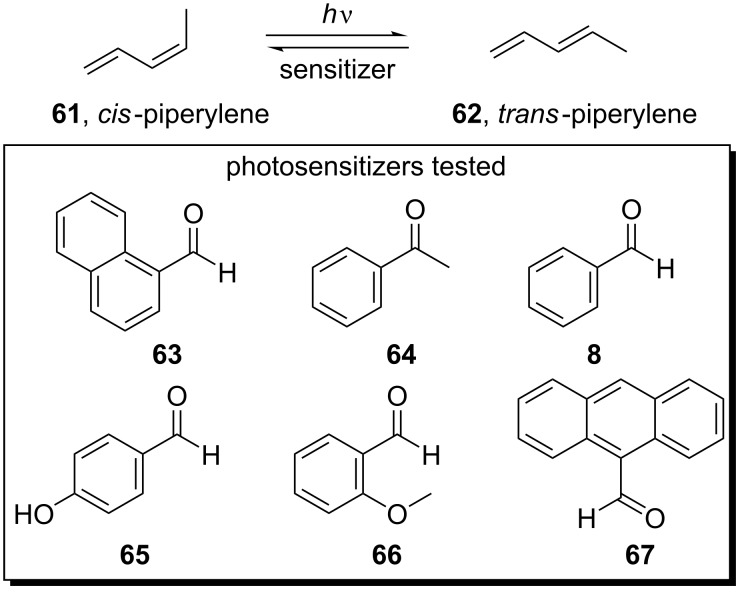
*ci*s/*trans* isomerization employing triplet states of photosensitizers.

In 1961, Hammond and co-workers studied the *cis*/*trans* isomerization of the piperylenes (1,3-pentadienes) **61** and **62** in the presence of carbonyl compounds as photosensitizers (Scheme 15) [50]. Among the compounds tested, 1-naphthaldehyde (**63**) provided a photostationary mixture rich in the *trans*-diene (*trans*/*cis* ratio ≈ 13). A year later, the same group further studied the *cis*/*trans* isomerization of the piperylenes **61** and **62** utilizing energy transfer from the triplet states of various aldehydes and ketones that were used as photosensitizers (Scheme 15) [51].

**Scheme 16:**
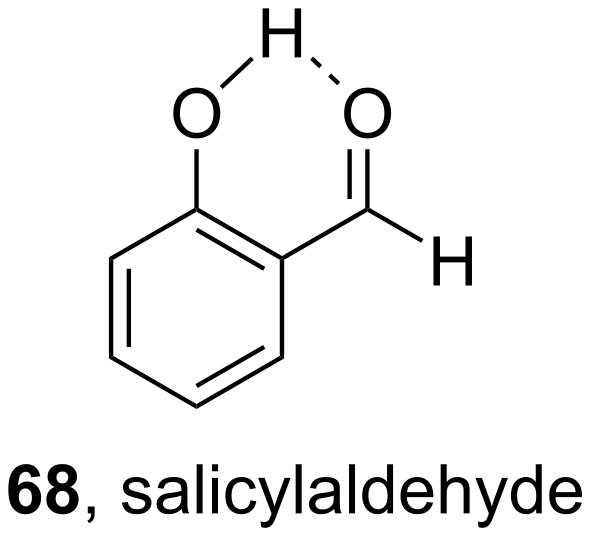
Salicylaldehyde (**68**) forms an internal hydrogen bond.

In more detail, a solution of *cis*-piperylene (**61**) in benzene in the presence of various carbonyl compounds was irradiated using a Hanovia quartz immersion reactor. It was shown that compounds that had a triplet state energy of 70 kcal/mol or more produced photostationary mixtures of a *trans*/*cis* ratio of ≈1.25. Such compounds were acetophenone (**64**), benzaldehyde (**8**), 4-hydroxybenzaldehyde (**65**), and 2-methoxybenzaldehyde (**66**). However, 9-anthraldehyde (**67**), which has a very low-lying triplet state below 50 kcal/mol, proved to be completely inactive as the photosensitizer. Also, no reaction was observed when salicylaldehyde (**68**) was employed as a photosensitizer, despite of a triplet state energy of ≈70 kcal/mol, most probably due to an internal hydrogen bond (Scheme 16).

**Scheme 17:**
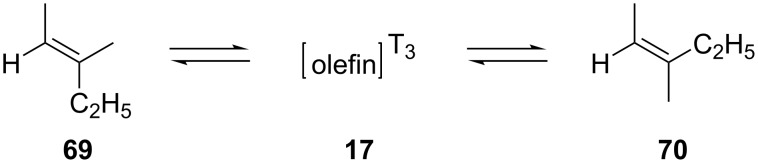
Olefin isomerization via energy transfer from a carbonyl compound.

As mentioned before, in the section on the photophysical properties of aldehydes, in 1968, Yang and co-workers investigated the mechanism of energy transfer from the triplet states of carbonyl compounds to simple olefins [15–16]. In more detail, in order to find out if the excitation energy of the triplet state of the carbonyl compounds could be transferred to the olefins in the Paterno–Büchi reaction, they performed a photochemical reaction between benzaldehyde (**8**) or benzophenone and the 3-methyl-2-pentenes **69** and **70**, respectively. The isomerization of the starting olefins indicated a bimolecular energy transfer mechanism (Scheme 17).

**Scheme 18:**
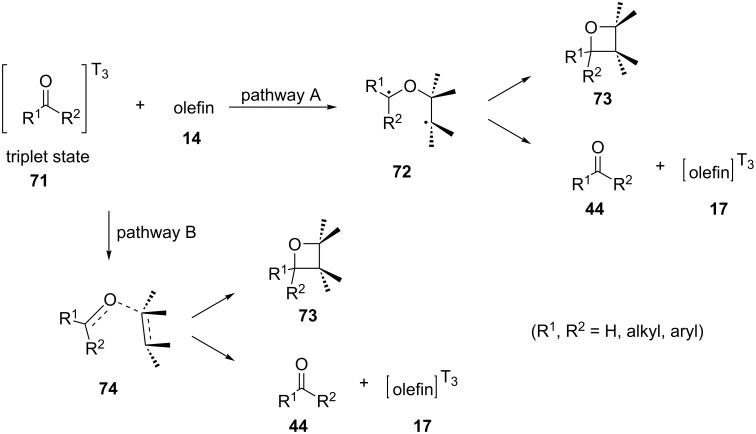
Mechanistic pathways for the Paterno–Büchi reaction.

Benzophenone proved to transfer the triplet state energy more efficiently than benzaldehyde (**8**). Furthermore, the triplet state of the carbonyl compound was thought to be the intermediate for both the Paterno–Büchi oxetane formation and the olefin isomerization. Two possible mechanistic pathways for the Paterno–Büchi reaction were described. The first one included an attack on the π-system of the olefin by the oxygen atom of the excited carbonyl compound, forming a biradical intermediate **72**. Then, the biradical intermediate **72** could either cyclize to produce an oxetane **73** or dissociate to form again the carbonyl compound **44** and a triplet state olefin molecule **17** in a nonplanar configuration. Both processes, oxetane formation and isomerization, were thought to proceed via the triplet state of benzaldehyde. The alternative pathway includes a polarization of the π-system of the olefin, forming a transition state intermediate **74** where the olefin moiety is no longer planar (Scheme 18).

**Scheme 19:**
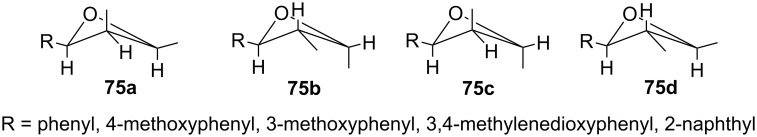
Isomeric oxetanes formed after photochemical addition of aryl aldehydes to 2-butenes.

**Scheme 20:**
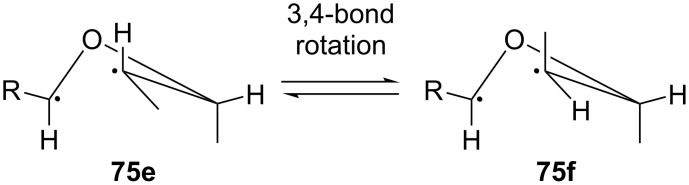
Rotation of the C3–C4 bond of the biradical intermediate may lead to all four conformations.

In 1973, the same group investigated the photochemical addition of benzaldehyde (**8**), 4-methoxybenzaldehyde, 3-methoxybenzaldehyde, 3,4-dimethylenedioxybenzaldehyde, and 2-naphthaldehyde to isomeric 2-butenes after irradiation using a Hanovia 450 W mercury arc [17]. If the reaction proceeded stereospecifically, *cis*-2-butene would have led to the formation of the oxetanes **75a** and **75b**, while *trans*-2-butene would have led to the oxetanes **75c** and **75d** (Scheme 19). It was suggested that the reaction proceeded through a long-lived biradical intermediate and rotation of the C3–C4 bond could in principle lead to all four possible oxetanes (Scheme 20). However, the formation of the oxetanes **75a** and **75d** was favored over that of the oxetanes **75b** and **75c**, probably because the intermediates for the products **75a** and **75d** contained the residue group and the 4-methyl group at the favorable pseudoequatorial position.

**Scheme 21:**
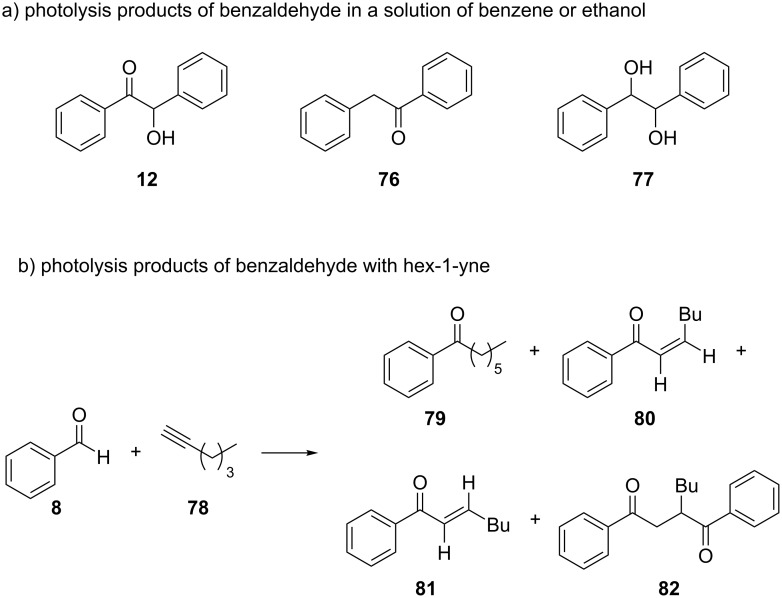
Photolysis products of benzaldehyde (**8**) in different solvents. a) In benzene or ethanol. b) In hex-1-yne.

In 1972, Bradshaw and co-workers studied the photolysis products of benzaldehyde (**8**) in different solvents [52]. The irradiation of benzaldehyde (**8**) in a benzene solution initially led to benzoin (**12**) formation, but after 14 hours of irradiation, the compounds **12**, **76**, and **77** were obtained in equal amounts. The irradiation of benzaldehyde (**8**) in ethanol yielded mostly compound **77** and traces of **12** (Scheme 21a). When benzaldehyde (**8**) was mixed with hex-1-yne (**78**), after a short irradiation time, the main products that were obtained were **80**, **81**, and **82**, as shown in Scheme 21b. The authors claimed that the compounds **80** and **81** could have formed by the addition of a benzoyl radical (**10**) to hex-1-yne (**78**). The compound **79** could have occurred as a reduction product of **80** and **81**. Finally, the compound **82** could have resulted from the addition of the benzoyl radical (**10**) to **80** and **81**.

**Scheme 22:**
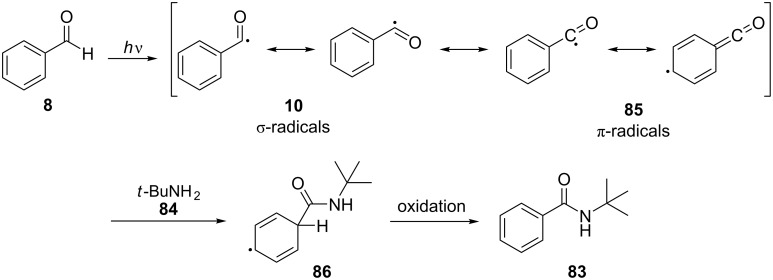
*N*-*tert*-Butylbenzamide formation proceeds via a benzoyl radical.

In 1976, Davidson and co-workers reported the formation of *N*-*tert*-butylbenzamides, such as **83**, from *tert*-butylamine (**84**) and substituted aromatic aldehydes, such as **8** [53]. The reaction between the substituted benzaldehydes and *tert*-butylamine (**84**) took place in a benzene solution by being irradiated for 16 h using a 100 W Hanovia medium-pressure mercury lamp. The aromatic aldehydes tested included benzaldehyde (**8**), 4-chlorobenzaldehyde, 2,4-dichlorobenzaldehyde, and 4-fluorobenzaldehyde, which gave the corresponding benzamides in moderate to low yield. The reaction mechanism was thought to proceed via the benzoyl radical (**10**) formed after the irradiation of **8**. The benzoyl π-radical **85** formed could then react with the nucleophilic *tert*-butylamine (**84**), and the radical intermediate **86** could be rapidly oxidized, either by other radicals or by oxygen, to the amide **83**, as shown in Scheme 22.

The low yield of the amide formation was attributed mainly to a concurrent imine formation, which was catalyzed by benzoic acid formed as a result of benzaldehyde (**8**) oxidation during the photoreaction. There was also a competition between two bimolecular processes, the attack of a triplet state aldehyde molecule on a ground state aldehyde molecule and the reaction of a triplet state aldehyde molecule with the amine.

**Scheme 23:**
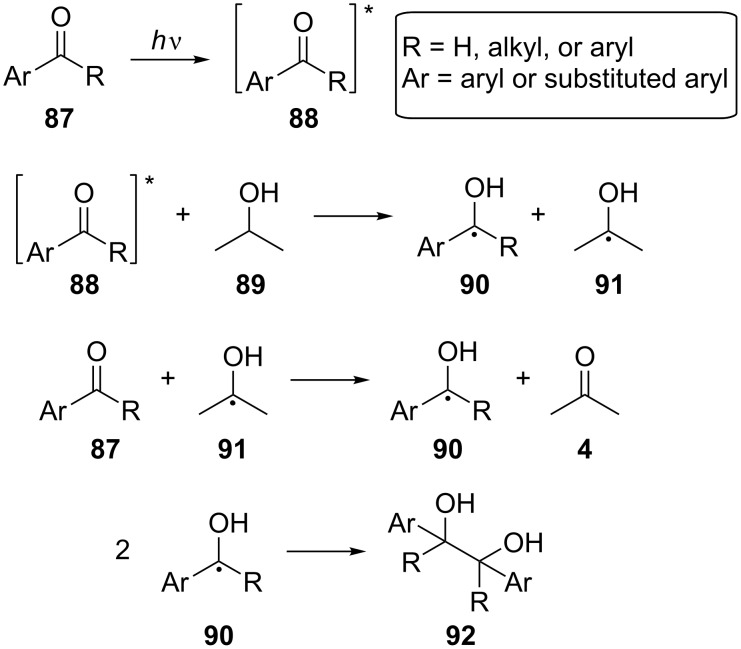
Photochemical pinacol coupling.

In 2003, Li and co-workers developed a novel method for the pinacol reaction, the coupling of aromatic aldehydes and ketones, using solar energy [54]. The aromatic carbonyl compounds were dissolved in isopropanol and exposed to direct sunlight for 7–10 days to give the corresponding 1,2-diols **92** in high to moderate yield. The excitation of the carbonyl compound **87** was followed by hydrogen atom abstraction from the solvent **89**, affording the α-hydroxybenzyl radical **90**, which then coupled, forming the 1,2-diol **92** (Scheme 23).

**Scheme 24:**
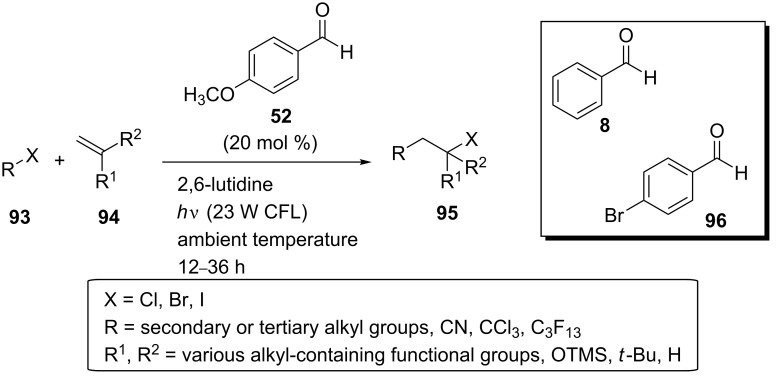
Photochemical ATRA catalyzed by 4-anisaldehyde (**52**).

In 2014, Melchiorre and co-workers found that 4-anisaldehyde (**52**) could efficiently catalyze the intermolecular atom transfer radical addition (ATRA) of the haloalkanes **93** to the olefins **94** under irradiation with a household 23 W compact fluorescent light (CFL) bulb at ambient temperature [55]. A base, 2,6-lutidine, was used to neutralize traces of HX (X = halogen), which formed by the homolytic cleavage of the C–X bond of the haloalkanes **93** (Scheme 24).

**Scheme 25:**
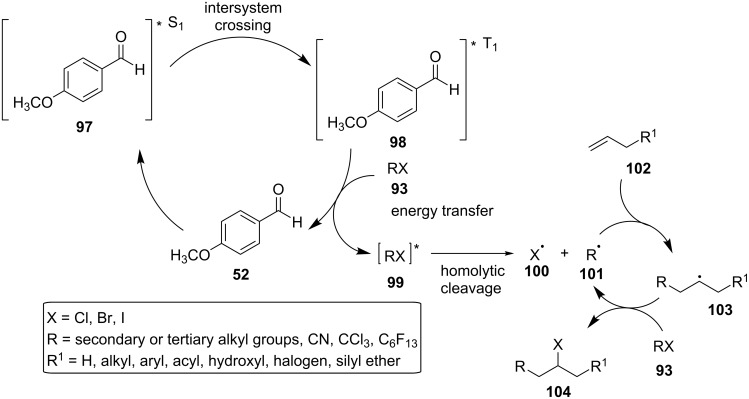
Proposed triplet sensitization mechanism of the ATRA reaction in the presence of 4-anisaldehyde (**52**).

Among the aldehydes tested, benzaldehyde (**8**) and 4-bromobenzaldehyde (**96**) were also effective as photocatalysts, providing, however, a lower product yield. On the contrary to aromatic aldehydes, benzophenone, which was also tested as a photocatalyst, could promote the reaction only when used in superstoichiometric amounts. This way, 4-anisaldehyde (**52**) was found to be the most effective organocatalyst. In the absence of light or 4-anisaldehyde (**52**), no transformation was observed. No transformation was observed either in the presence of 2,2,6,6-tetramethylpiperidine-1-oxyl, 2,6-di-*tert*-butyl-4-methylphenol or air, indicating a radical mechanism. The reaction proceeded efficiently for a wide range of substrates in moderate to excellent yield, including various alkyl halides **93**, carbon tetrachloride, 2-norbornene, cyclic alkenes, a terminal disubstituted olefin, and a terminal alkyne. The reaction mechanism was thought to proceed via energy transfer from the aldehyde catalyst to the haloalkanes. Using cutoff filters for certain wavelengths, the authors found that the near-UV part of the CFL emission was necessary for the excitation of 4-anisaldehyde (**52**) through a n→π* transition. No ground state association between the reaction components was detected. Moreover, the excited 4-anisaldehyde (**98**) underwent energy transfer over the addition to the double bond of the alkene, as no Paterno–Büchi cycloadducts were observed. Furthermore, the detection of the photolysis products of the alkyl halides mentioned above indicated the presence of triplet state 4-anisaldehyde (**98**), which possessed a relatively long lifetime and an energetic value sufficient for C–X bond cleavage. The participation of the triplet state 4-anisaldehyde (**98**) in the reaction mechanism was also confirmed by the complete inhibition of the ATRA reaction in the presence of oxygen, a triplet state quencher, as well as by the lowered rate of the reaction in the presence of the triplet energy quencher 2,5-dimethylhexa-2,4-diene or pyridazines, additives with lower triplet state energies than 4-anisaldehyde (**52**). The fact that the solvent polarity did not affect the sensitivity of the process supported the energy transfer pathway. Thus, the proposed triplet sensitization mechanism of the photocatalytic ATRA reaction is depicted in Scheme 25.

**Scheme 26:**
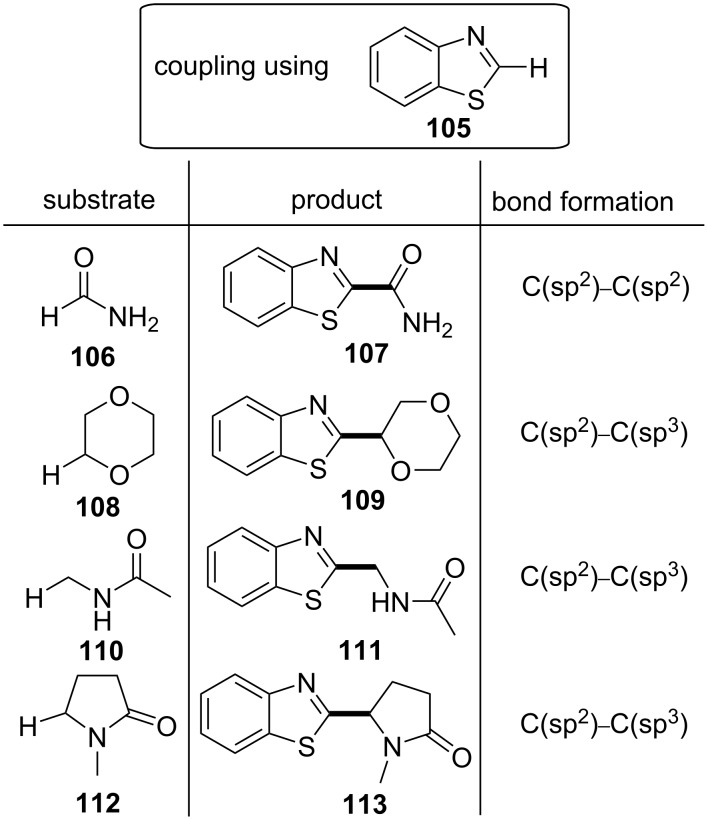
Benzaldehyde-mediated photoredox CDC reaction: compatible amides and ethers.

In 2016, Ji and co-workers developed a new photoredox cross-dehydrogenative coupling (CDC) method for the α-heteroarylation of amides (α to nitrogen, e.g., formamide) and ethers through C–H activation using various five- and six-membered heteroarenes (e.g., benzothiazole) and employing benzaldehyde (**8**) as the photoinitiator [56]. This protocol was compatible with both C(sp^3^)–H activation (*N*-alkyl C–H bonds of amides or C_α_–H bonds of ethers) and C(sp^2^)–H activation (carbonyl C–H bonds of formamides). Some of the amides or ethers found to be compatible with this method are shown in Scheme 26.

**Scheme 27:**
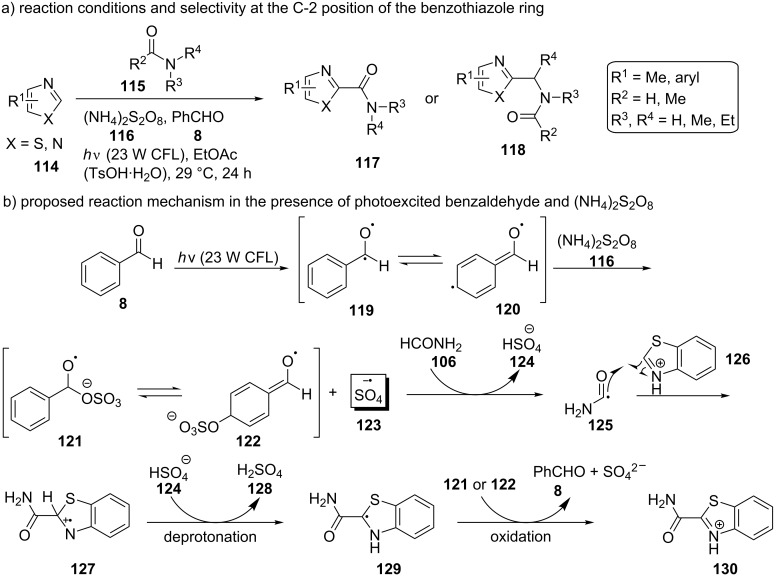
Photoredox cross-dehydrogenative coupling (CDC) conditions and proposed reaction mechanism.

A wide range of heteroarenes **114** was also found compatible with this method, including substituted benzothiazole substrates, substituted benzimidazoles, and thiazoles, giving selectively the C-2-substituted product in moderate to excellent yield (Scheme 27a). However, only traces of the product were detected employing benzoxazole. Six-membered heteroaromatic compounds, such as pyridine, pyrazine, quinazoline, and even carboline, in some cases fused to a benzene ring, were also found to be compatible. 23 W CFL bulbs were used as the light source for the excitation of benzaldehyde. (NH_4_)_2_S_2_O_8_ (**116**) was used as the radical initiator. Photoexcited benzaldehyde (**8**) enhanced the (NH_4_)_2_S_2_O_8_ (**116**) decomposition, accompanied by the generation of the sulfate radical **123** (Scheme 27b). The sulfate radical then reacted with formamide (**106**) to produce the carbamoyl radical **125**, which could perform a nucleophilic addition to the C-2 position of the protonated benzothiazole **126**. A deprotonation, followed by an oxidation, most probably by the intermediates **121** and **122**, could lead to the formation of the desired product and the regeneration of benzaldehyde (**8**). In most cases, *p*-toluenesulfonic acid (TsOH) was also used in order to improve the yield through the protonation and activation of benzothiazole. The proposed reaction mechanism, as explained above, is depicted in Scheme 27b.

Even 0.5 equivalents of benzaldehyde were able to promote the reaction. Furthermore, atmospheric oxygen and TEMPO inhibited the reaction. These facts suggest a radical mechanism. A very low yield was observed in the absence of irradiation. No product was detected in the absence of (NH_4_)_2_S_2_O_8_ (**116**), indicating the necessity of the benzaldehyde (**8**)-mediated decomposition of (NH_4_)_2_S_2_O_8_ (**116**) to generate carbamoyl radicals **125**. Varying the light wavelength, the authors observed that it was the near-UV region that was required for the reaction. There was no ground state association observed between the reaction components. Although the authors found that **8** went through an excited n→π* transition, it was indicated that the triplet state of benzaldehyde, **9**, was not involved since it would have been quenched by the acid. Thus, the excited state of benzaldehyde directly promoted the (NH_4_)_2_S_2_O_8_ (**116**) decomposition, initiating the radical chain reaction. Other substituted benzaldehydes were also investigated. Finally, experiments employing benzaldehyde-α-d_1_, showing no hydrogen/deuterium exchange, indicated no formation of a benzoyl radical (**10**) in this reaction.

**Scheme 28:**
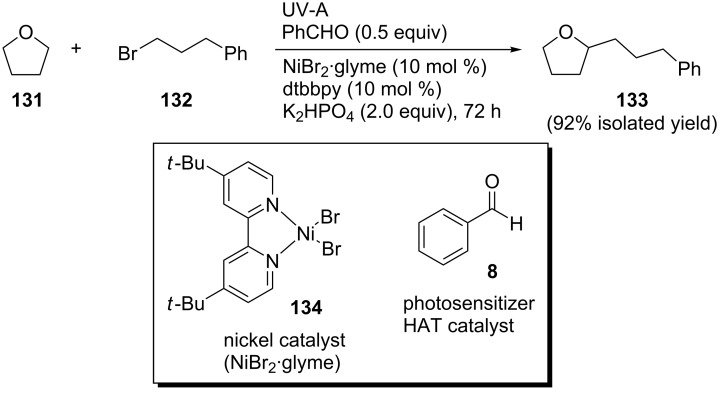
Optimized conditions for the photoredox merger reaction.

In 2019, Hashmi and co-workers reported a selective photoredox merger C(sp^3^)–H alkylation/arylation of ethers using benzaldehyde as the photoorganocatalyst and nickel as the transition metal catalyst [57]. For the coupling between (3-bromopropyl)benzene (**132**) and THF (**131**), presented in Scheme 28, NiBr_2_·glyme (**134**) was employed as the precatalyst, 4,4’-di-*tert*-butyl-2,2’-bipyridyl (dtbbpy) as the ligand for the nickel catalyst, benzaldehyde (**8**) as the photosensitizer and hydrogen abstractor, K_2_HPO_4_ as the base, and UV-A light for the excitation of **8**. The reaction mixture was placed under a N_2_ atmosphere and irradiated for 72 h.

**Scheme 29:**
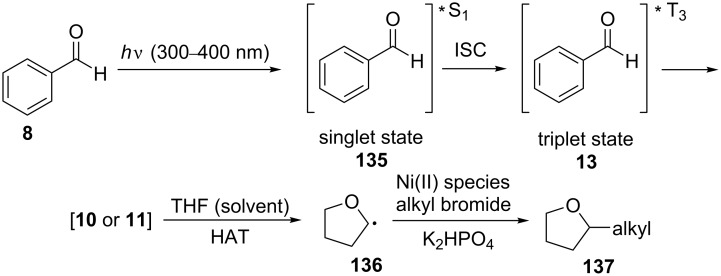
Proposed mechanism for the C(sp^3^)–H alkylation/arylation of ethers.

Their hypothesis was that the excitation of benzaldehyde (**8**) to the singlet state followed by ISC to the triplet state could lead to a HAT process from the THF solvent molecule, resulting in the α-hydroxybenzyl radicals **10** or **11** and solvent radicals, which could then undergo coupling with an alkyl bromide in the presence of the nickel catalyst and a base via a typical oxidative addition, insertion, and reductive elimination sequence to afford the desired product (Scheme 29).

**Scheme 30:**
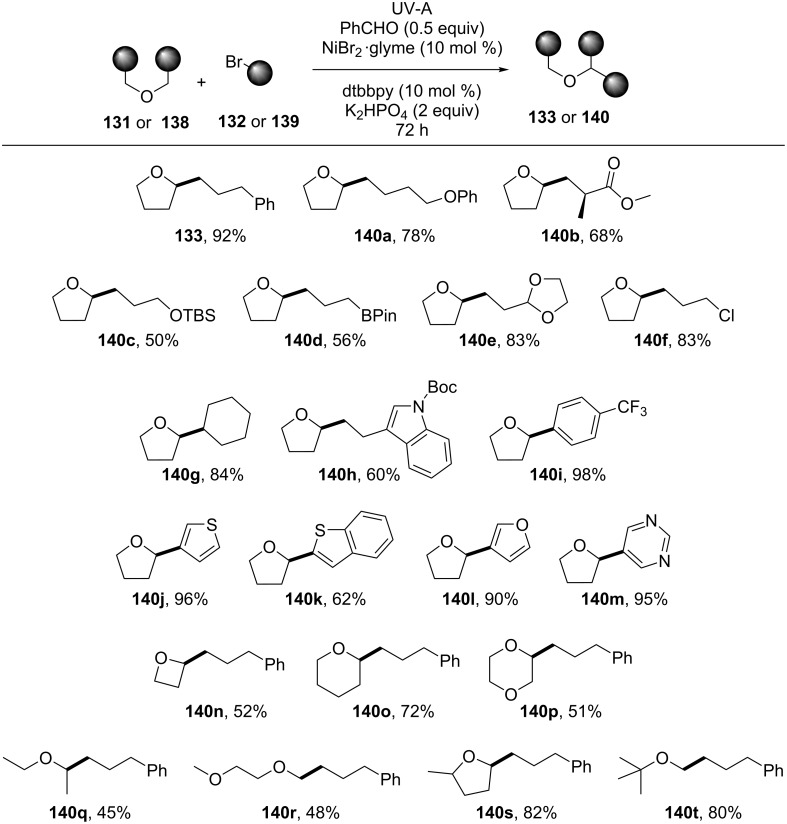
Substrate scope for the photochemical alkylation of ethers.

In the absence of K_2_HPO_4_, only traces of the desired product were detected. Other known photosensitizers, such as acetone (**4**), acetophenone (**64**), benzophenone, as well as substituted benzaldehydes were not as efficient as benzaldehyde (**8**). Using 4-anisaldehyde (**52**) and irradiation with CFL bulbs, no product was obtained. The reaction could also take place in acetone as the solvent using 10 equivalents of THF and applying irradiation for 96 h. The aryl (i.e., **133**), ether (i.e., **140a**), ester (i.e., **140b**), *tert*-butyldimethylsilyloxy (i.e., **140c**), boronic ester (i.e., **140d**), acetal (i.e., **140e**), and indol products (i.e., **140h**) of various bromo-substituted starting materials were successfully obtained. For 1-bromo-3-chloropropane, the coupling occurred selectively at the carbon atom bearing the bromine atom, yielding **140f**. The products **140g**, **140i**, and **140j**–**m** of cyclic, aryl, and heteroaryl bromides, respectively, were also found compatible with this methodology. All bromides mentioned above afforded the desired products in good to excellent yields. However, benzyl and vinyl bromides did not prove efficient for this coupling reaction. Common cyclic and acyclic ethers, symmetric or nonsymmetric, such as oxetane, tetrahydropyran, dioxane, 2-methyltetrahydrofuran, and 2-methoxy-2-methylpropane, were found compatible, affording the desired products **140n**–**t** in moderate to good yields (Scheme 30).

**Scheme 31:**
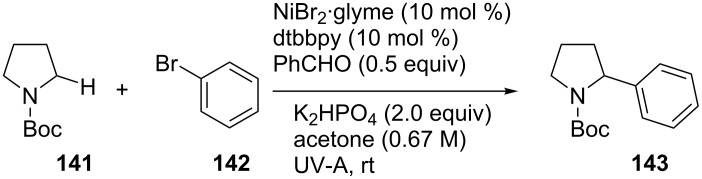
C(sp^3^)–H Functionalization of N-containing molecules.

A few months later, the same research group extended this methodology to an α-C(sp^3^)–H bond functionalization of nitrogen-containing molecules and thioethers [58]. In order to achieve the highest yield, an optimum concentration of acetone (**4**) was necessary since at higher concentrations, the nickel catalyst would not dissolve completely. By increasing the amount of **141**, the yield gradually increased, so that 5 equivalents of *N*-Boc-pyrrolidine (**141**) were used, prolonging the irradiation time to 60 h, to achieve a yield of 88%. Other nickel salts were also tested, but they did not prove to be more efficient than the one already used. The optimized reaction conditions are presented in Scheme 31.

**Scheme 32:**
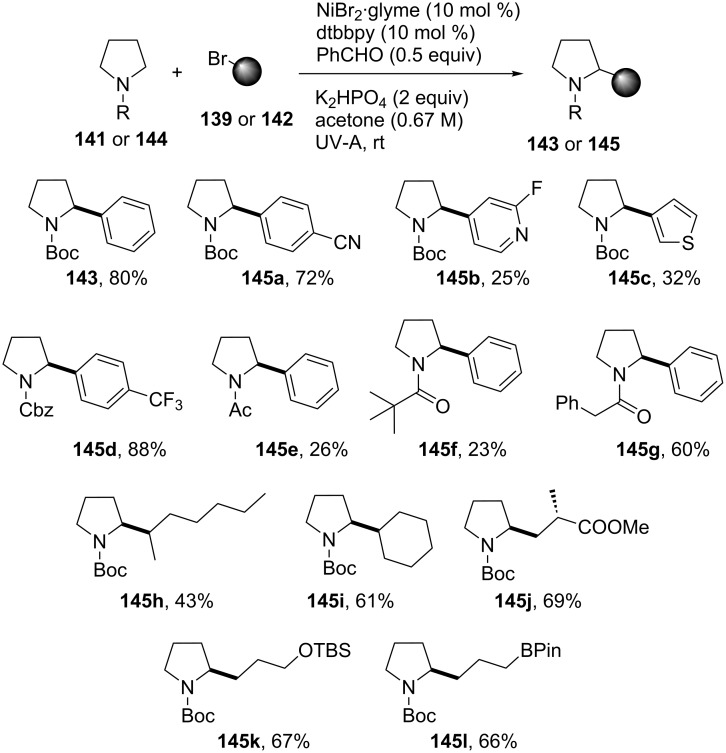
Substrate scope for the photochemical alkylation of N-containing molecules.

**Scheme 33:**
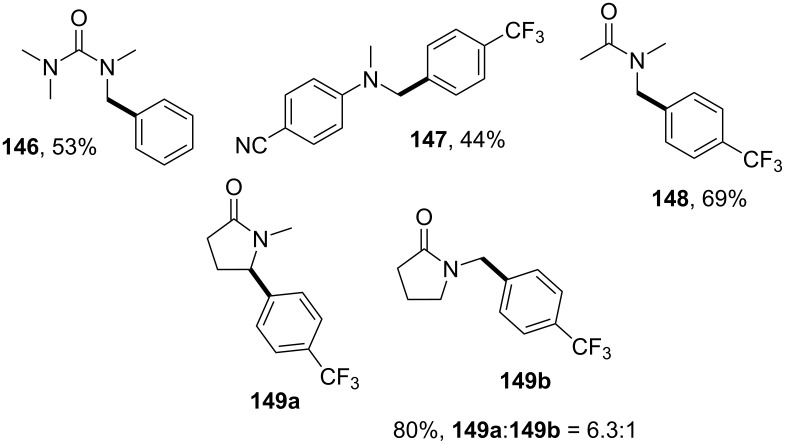
Additional products yielded by the photochemical alkylation reaction of N-containing molecules.

A wide variety of aryl bromides, containing either electron-donating or electron-withdrawing substituents, was found to be compatible with this methodology, affording the products **143** and **145a**–**d** in good yield. The N-substituted pyrrolidines **144a**/**d**–**g** were also compatible, providing the desired products in good yields, except from the *N*-acetyl (**144e**) and *N*-pivaloylpyrrolidines (**144f**), for which the yields dropped considerably. The alkylation of the pyrrolidine **141** was also possible employing a variety of alkyl bromides, including cyclic and acyclic secondary alkyl bromides as well as alkyl bromides carrying functional groups such as organosilyl and boronate moieties. However, the solvent had to be changed from acetone to acetonitrile in order to achieve good yields of **145h**–**l** (Scheme 32). Additionally, products derived from ureas (i.e., **146**), *N*,*N*-dimethylaniline (i.e., **147**), amides (i.e., **148**), and lactams (i.e., **149a**) were obtained. Moreover, for *N*-methylpyrrolidinone, α-arylation was achieved in a good yield and regioselectivity for the substitution of the 2-position, yielding **149a**, rather than for the *N*-methyl group, which would yield **149b** (Scheme 33).

**Scheme 34:**
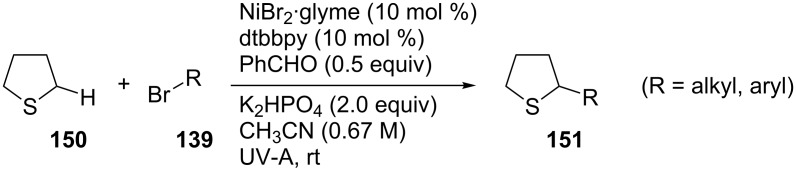
C(sp^3^)–H functionalization of thioethers.

In the same manner, thioethers were also susceptible to α-arylations or alkylations, using bromides containing a variety of functional groups, giving moderate to good yields (Scheme 34).

**Scheme 35:**
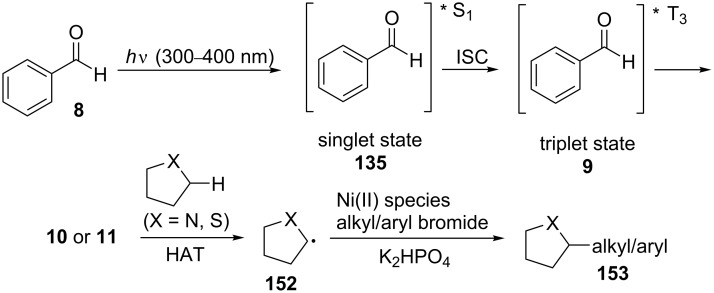
Proposed mechanism for the C(sp^3^)–H alkylation/arylation of N-containing molecules and thioethers.

The proposed mechanism for the C(sp^3^)–H functionalization of N-containing molecules and thioethers is in accordance with the one described previously for ether functionalizations and is presented in Scheme 35.

**Scheme 36:**
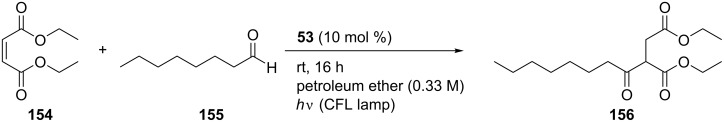
Hydroacylation using 4-cyanobenzaldehyde (**53**) as the photoinitiator.

In 2019, Kokotos and co-workers employed 4-cyanobenzaldehyde (**53**) as the photoinitiator for the hydroacylation of Michael acceptors under irradiation with 80 W household bulbs [59]. 4-Cyanobenzaldehyde (**53**) exhibited the highest yield (86%) among 25 substituted benzaldehydes in the reaction between diethyl maleate (**154**) and octanal (**155**) (Scheme 36). During the optimization, different solvents, catalyst loadings, and aldehyde equivalents were tested. Petroleum ether (bp 40–60 °C) afforded a high yield even at 10 mol % catalyst loading. In the absence of light or a catalyst or when the reaction was placed in the dark at 60 °C, the reaction did not proceed.

**Scheme 37:**
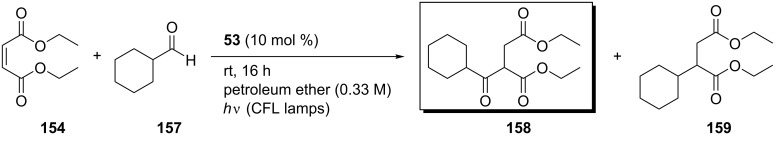
Selectivity for the formation of the α,α-disubstituted aldehydes.

When α,α-disubstituted aldehydes, e.g., cyclohexyl carboxaldehyde (**157**) are used in this type of reaction, the acyl radicals formed can easily be decarbonylated due to the rather stable secondary radical formed, so the selectivity of the reaction was studied. For this, the authors studied the reaction between diethyl maleate (**154**) and cyclohexyl carboxaldehyde (**157**) to find that 4-cyanobenzaldehyde (**53**) again proved to be the best photoinitiator in terms of the yield and selectivity, affording the desired carbonylated product **158** in a selectivity ratio of >20:1 (Scheme 37).

**Scheme 38:**
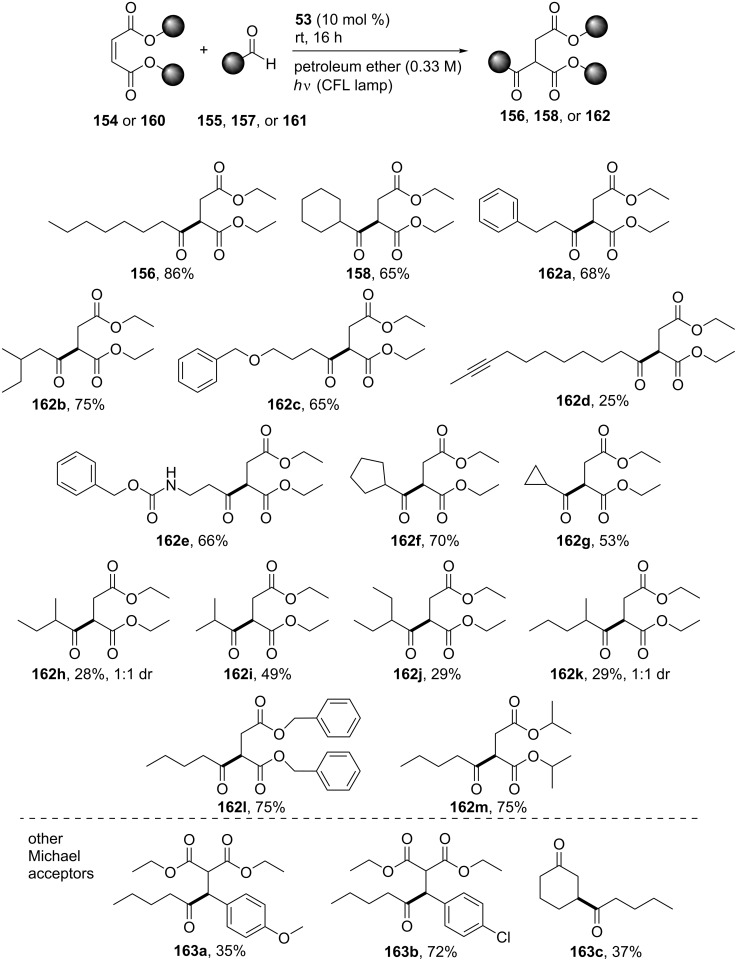
Substrate scope for the photochemical addition of aldehydes to Michael acceptors.

Primary alkyl aldehydes, aldehydes containing branched alkyl chains or cyclic aliphatic moieties, amide bonds, triple bonds, or ether moieties afforded the desired products in moderate to good yield (**156**, **158**, **162a**–**e**). α,α-Disubstituted aldehydes also afforded the products in good yield and moderate to good selectivity (**162f**–**k**). Benzyl diesters and aliphatic diesters were compatible with this methodology, affording the products **162l** and **162m** in moderate to good yield, while the products deriving from α,β-unsaturated carbonyl compounds, the products **163a**–**c** were obtained in moderate yield (Scheme 38).

**Scheme 39:**
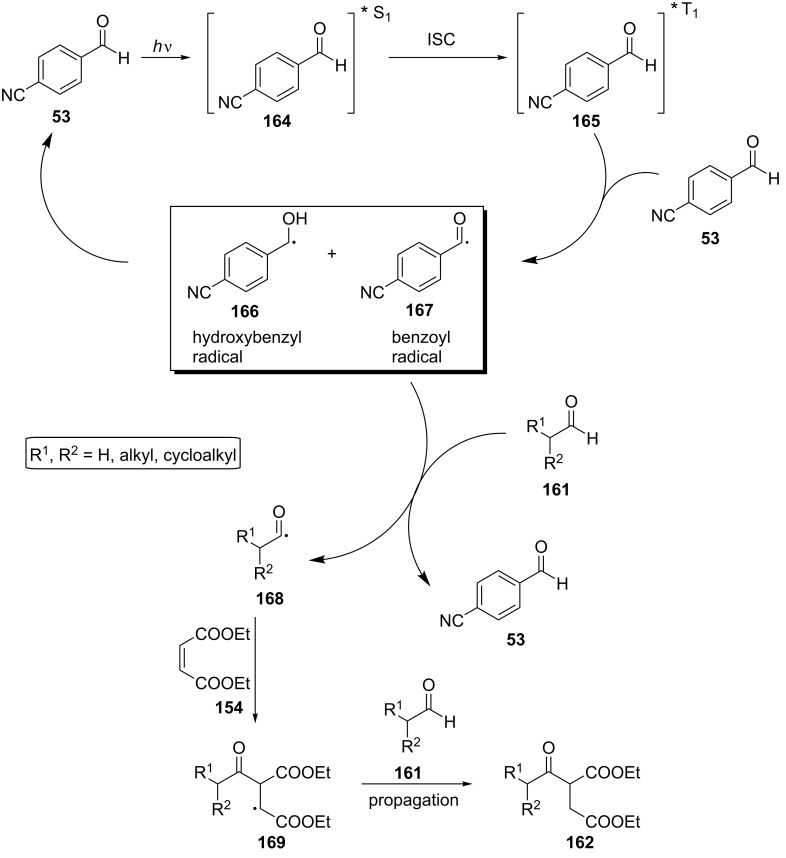
Proposed mechanism for the hydroacylation of Michael acceptors using 4-cyanobenzaldehyde (**53**) as the photoinitiator.

Next, the authors conducted various mechanistic experiments, including fluorescence quenching studies, UV–vis spectroscopy experiments, calculation of the quantum yield, a cutoff experiment below 400 nm, GC–MS analysis, and NMR spectroscopic mechanistic experiments. They also concluded that an energy transfer mechanism could not be possible since the solvent employed affected the yield. The drop in the yield was insignificant in the absence of oxygen. An insignificant drop in the yield was also noticed when an electron scavenger, CuCl_2_, was added to the reaction mixture, excluding a single electron transfer process. When a triplet state quencher, anthracene, was added, the reaction did not proceed, indicating that the triplet state of 4-cyanobenzaldehyde (**53**) was involved. Radical traps also inhibited the reaction, indicating a radical propagation mechanism. Based on these observations, they proposed a possible mechanistic pathway (Scheme 39). Under irradiation, 4-cyanobenzaldehyde (**53**) could transit to the excited singlet state **164** and then to the triplet state **165** through intersystem crossing. The interaction with a ground state molecule of **53** may lead to the radical pair of the hydroxybenzyl and benzoyl radicals **166** and **167**. The interaction of the benzoyl radical **167** with the aldehyde **161** could afford the acyl radical **168** through a HAT process, which then could add to diethyl maleate (**154**) and afford the desired product **162** through a propagation mechanism.

**Scheme 40:**
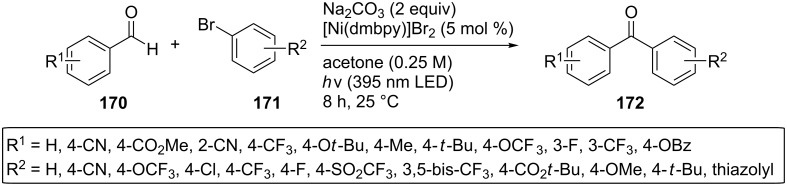
Catalytic arylation of aromatic aldehydes by aryl bromides in which the reaction product acts as the photocatalyst.

In 2019, König and co-workers presented a catalytic arylation of aromatic aldehydes **170** by aryl bromides **171** using UV as the light source and a nickel catalyst [60]. The authors suggested that the product **172**, a substituted benzophenone, could act as the photocatalyst and the hydrogen atom transfer agent in this reaction (Scheme 40). They optimized the reaction conditions with regard to the nickel catalyst, the solvent, the base, and the irradiation wavelength. Next, they investigated the reaction potential, with most aryl bromides and benzaldehydes tested being compatible to this transformation, affording the products in moderate to excellent yield.

**Scheme 41:**
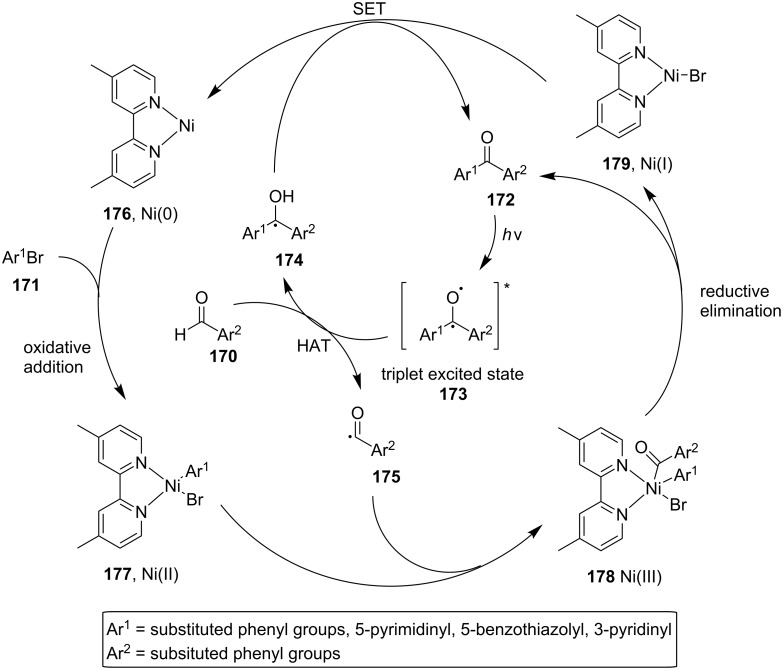
Proposed mechanism for the catalytic arylation of benzaldehydes by aryl bromides in which the reaction product acts as a photocatalyst.

The proposed photocatalytic cycle starts with the excitation of the benzophenone **172** to the triplet state **173** by UV irradiation, followed by HAT from the aldehyde **170** to **173** and the formation of the acyl radical **175**. At the same time, the nickel(0) complex **176** performs an oxidative addition reaction to the aryl bromide **171**, and the nickel(II) complex **177** formed reacts with an acyl radical **175** to give the nickel(III) complex **178**, which could then undergo a reductive elimination reaction to furnish the desired ground state benzophenone product **172**. The nickel(0) catalyst can then be regenerated by SET (Scheme 41). However, they suggested that the first photocatalytically active species can be initiated by the reaction mixture, containing traces of different benzaldehyde photolysis products together with benzaldehyde.

**Scheme 42:**
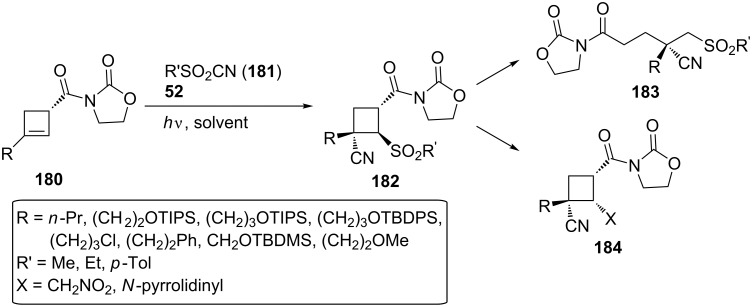
Functionalization of the chiral cyclobutanes **180**.

In 2020, Landais and co-workers developed a photosensitized sulfonylcyanation of chiral cyclobutenes [61]. Although cyclobutanes are substrates of significant pharmaceutical use, the unsaturated analogs, cyclobutenes, are characterized by a poor reactivity of the π-bond, restricting the access to chiral cyclobutanes. This research group worked on a photocatalyzed addition of an electrophilic sulfonyl radical and a cyanide group across the π-bond of a chiral cyclobutene **180**, providing highly functionalized cyclobutanes **182** and an access to the enantioenriched cyanosulfones **183**, resulting from cyclobutane ring opening, or the new tetrasubstituted cyclobutanes **184** (Scheme 42).

**Scheme 43:**
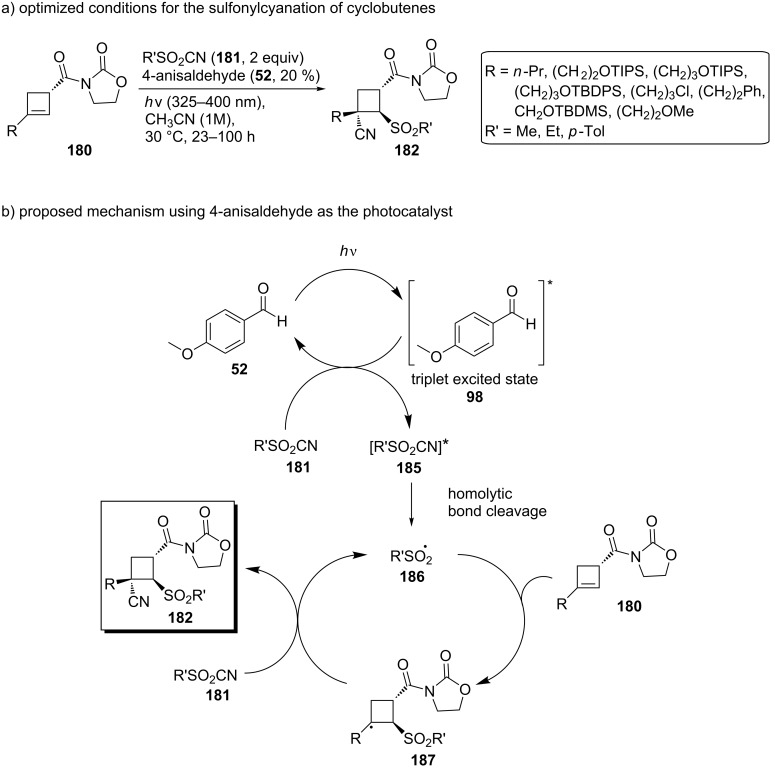
Optimized reaction conditions and proposed mechanism for the sulfonylcyanation of cyclobutenes.

The cyclobutenes **180** were subjected to a photocatalyzed radical addition, and upon optimization, 4-anisaldehyde (**52**) proved to be a better photocatalyst under UV-A irradiation than the initially used eosin Y. Compound **182**, the all-*trans*-isomer, was the major product. The optimized conditions are presented in Scheme 43a. Conducting several control experiments and noticing that the presence of 4-anisaldehyde (**52**), UV-A light, and the absence of a triplet state quencher were essential for this transformation, they proposed a possible reaction mechanism (Scheme 43b): Energy transfer from triplet-excited-state 4-anisaldehyde (**98**) to the sulfonyl cyanide **181** can lead to a homolytic cleavage of the C–S bond, providing the sulfonyl radical **186**. The sulfonyl radical **186** can then approach *anti* to the oxazolidinone moiety in **180**, generating a β-sulfonyl radical **187**, which, once trapped by another sulfonyl cyanide molecule **181**, affords the desired product **182**, and the sulfonyl radical reenters the catalytic cycle.

Various alkyl substituents on the cyclobutene ring, such as substituents bearing a chlorine atom, or silyl ethers were well-tolerated. However, bulky groups (e.g., TBDMS) led to lower yields. Methyl, ethyl, or aryl sulfones were all compatible with this methodology, and could even be extended to functionalized sulfonyl cyanides, though providing lower yields. In every case, the diastereoselectivity was high (dr > 19:1:1) and in favor of the all-*trans*-product.

## Conclusion

In this review, we summarized the photophysical properties of aromatic aldehydes and the potential that (aromatic and aliphatic) aldehydes have as photoinitiators. In more detail, we cited their use as initiators in polymerization and photografting reactions and finally, we provided the applications of aldehydes as photoinitiators in organic synthesis. We strongly believe that as researchers seek greener and sustainable processes to advance chemistry, the use of aldehydes in conjunction with light irradiation, even sunlight, will become more popular, providing a vast array of applications.

## References

[1] Prier C K, Rankic D A, MacMillan D W C (2013). Chem Rev.

[2] Skubi K L, Blum T R, Yoon T P (2016). Chem Rev.

[3] Cambié D, Bottecchia C, Straathof N J W, Hessel V, Noël T (2016). Chem Rev.

[4] Kärkäs M D, Porco J A, Stephenson C R J (2016). Chem Rev.

[5] Gad S C (2011). Device Safety Evaluation.

[6] Nicewicz D A, Nguyen T M (2014). ACS Catal.

[7] Ravelli D, Fagnoni M, Albini A (2013). Chem Soc Rev.

[8] Romero N A, Nicewicz D A (2016). Chem Rev.

[9] Ravelli D, Protti S, Fagnoni M (2016). Chem Rev.

[10] Sideri I K, Voutyritsa E, Kokotos C G (2018). Org Biomol Chem.

[11] Capaldo L, Ravelli D (2017). Eur J Org Chem.

[12] Cundall R B, Davies A S (1966). Trans Faraday Soc.

[13] Cocivera M, Trozzolo A M (1970). J Am Chem Soc.

[14] Closs G L, Paulson D R (1970). J Am Chem Soc.

[15] Yang N C, Cohen J I, Shani A (1968). J Am Chem Soc.

[16] Yang N C (1968). Photochem Photobiol.

[17] Yang N C, Kimura M, Eisenhardt W (1973). J Am Chem Soc.

[18] Berger M, Goldblatt I L, Steel C (1973). J Am Chem Soc.

[19] Atkins P W, Frimston J M, Frith P G, Gurd R C, McLauchlan K A (1973). J Chem Soc, Faraday Trans 2.

[20] Murai H, Obi K (1975). J Phys Chem.

[21] Ohmori N, Suzuki T, Ito M (1988). J Phys Chem.

[22] Silva C R, Reilly J P (1996). J Phys Chem.

[23] Long S R, Meek J T, Harrington P J, Reilly J P (1983). J Chem Phys.

[24] Frith P G, McLauchlan K A (1975). J Chem Soc, Faraday Trans 2.

[25] Metcalfe J, Brown R G, Phillips D (1975). J Chem Soc, Faraday Trans 2.

[26] Harriman A, Rockett B W, Poyner W R (1974). J Chem Soc, Perkin Trans 2.

[27] Defoin A, Defoin-Straatmann R, Kuhn H J (1982). J Labelled Compd Radiopharm.

[28] Defoin A, Defoin-Straatmann R, Kuhn H J (1984). Tetrahedron.

[29] Itoh T (1988). Chem Phys Lett.

[30] Zhu L, Cronin T J (2000). Chem Phys Lett.

[31] Bagchi A, Huang Y-H, Xu Z F, Raghunath P, Lee Y T, Ni C-K, Lin M C, Lee Y-P (2011). Chem – Asian J.

[32] Cui G, Lu Y, Thiel W (2012). Chem Phys Lett.

[33] Khudyakov I V, McGarry P F, Turro N J (1993). J Phys Chem.

[34] Fletcher K, Bunz U H F, Dreuw A (2016). ChemPhysChem.

[35] McGinniss V D, Provder T, Kuo C, Gallopo A (1978). Macromolecules.

[36] Suppan P (1975). J Chem Soc, Faraday Trans 1.

[37] Schuster D I, Goldstein M D, Bane P (1977). J Am Chem Soc.

[38] Aydın M, Arsu N (2006). Prog Org Coat.

[39] Allméar K, Hult A, Rårnby B (1988). J Polym Sci, Part A: Polym Chem.

[40] Liqun Z, Irwan G S, Kondo T, Kubota H (2000). Eur Polym J.

[41] Wang H, Brown H R (2004). J Polym Sci, Part A: Polym Chem.

[42] Zhao A, Li Z, Wang H (2010). Polymer.

[43] Wang H, Brown H R (2004). Macromol Rapid Commun.

[44] Wang H, Brown H R, Li Z (2007). Polymer.

[45] Han J, Wang H (2009). J Appl Polym Sci.

[46] Song A, Zhao D, Rong R, Zhang L, Wang H (2011). J Appl Polym Sci.

[47] Guo R, Gao Y, Wu M, Wang H (2013). Polymer.

[48] Coutinho K, Saavedra N, Canuto S (1999). J Mol Struct: THEOCHEM.

[49] Ma W, Zhang X, Ma Y, Chen D, Wang L, Zhao C, Yang W (2017). Polym Chem.

[50] Hammond G S, Leermakers P A, Turro N J (1961). J Am Chem Soc.

[51] Hammond G S, Turro N J, Leermakers P A (1962). J Phys Chem.

[52] Bradshaw J S, Knudsen R D, Parish W W (1972). J Chem Soc, Chem Commun.

[53] Davidson R S, Edwards J, Warburton S K (1976). J Chem Soc, Perkin Trans 1.

[54] Li J-T, Yang J-H, Han J-F, Li T-S (2003). Green Chem.

[55] Arceo E, Montroni E, Melchiorre P (2014). Angew Chem, Int Ed.

[56] Zhang Y, Teuscher K B, Ji H (2016). Chem Sci.

[57] Zhang L, Si X, Yang Y, Zimmer M, Witzel S, Sekine K, Rudolph M, Hashmi A S K (2019). Angew Chem, Int Ed.

[58] Si X, Zhang L, Hashmi A S K (2019). Org Lett.

[59] Sideri I K, Voutyritsa E, Kokotos C G (2019). ChemSusChem.

[60] Schirmer T E, Wimmer A, Weinzierl F W C, König B (2019). Chem Commun.

[61] Pirenne V, Traboulsi I, Rouvière L, Lusseau J, Massip S, Bassani D M, Robert F, Landais Y (2020). Org Lett.

